# Clinical predictors for bloodstream infection in systemic lupus erythematosus

**DOI:** 10.1080/07853890.2026.2680341

**Published:** 2026-06-03

**Authors:** Mengmin Ye, Yunying He, Siting Yi, Pingjuan Liu, Chenfeng Zhao, Yili Chen, Liubing Li

**Affiliations:** ^a^ Department of Laboratory Medicine, The First Affiliated Hospital of Sun Yat-sen University, Guangzhou, China; ^b^ Yunkang School of Medicine and Health, Nanfang College, Guangzhou, China; ^c^ Center for Clinical Laboratories, GuiZhou Hospital of the First Affiliated Hospital, Sun Yat-sen University, Guiyang, China

**Keywords:** Systemic lupus erythematosus, bloodstream infection, clinical predictors

## Abstract

**Objective:**

This study aimed to investigate the distribution of pathogenic bacteria and identify clinical predictors associated with bloodstream infection (BSI) in patients with systemic lupus erythematosus (SLE).

**Methods:**

The experimental group included 43 SLE patients with BSI (SLE-BSI), who were diagnosed and treated at the First Affiliated Hospital of Sun Yat-sen University between October 2013 and June 2023. The control group consisted of 172 SLE patients without BSI (SLE-non-BSI), randomly selected at a 1:4 ratio and matched for age, gender, and ward with the case group. Logistic regression analyses of clinical data and laboratory indicators were used to identify independent clinical predictors for SLE-BSI.

**Results:**

The most common pathogens identified in SLE-BSI patients were *Staphylococcus aureus* (18.6%), *Salmonella spp.* (16.3%) and *Escherichia coli* (11.6%). Multivariate logistic regression analysis revealed that fever (OR = 4.079, 95% CI: 1.582–10.517, *p* = 0.004), myalgia (OR = 5.637, 95% CI: 1.363–23.318, *p* = 0.017), and glucocorticoid use (OR = 2.690, 95% CI: 1.041–6.948, *p* = 0.041) were independent clinical predictors associated with the occurrence of SLE-BSI.

**Conclusion:**

For SLE patients receiving glucocorticoid therapy, the presence of myalgia and fever should raise suspicion of BSI. The findings may help optimize the clinical utility of blood culture testing, thereby facilitating the early identification of BSI in SLE patients.

## Introduction

Systemic lupus erythematosus (SLE) is a chronic autoimmune disease characterized by abnormal activation of the immune system and multi-system involvement. Patients typically experience a relapsing and remitting course of disease [1,2], and often present with nonspecific systemic symptoms such as fever, fatigue, and weight loss. The most characteristic clinical manifestations include skin and mucosal lesions like facial butterfly rash, photosensitivity, and oral ulcers. Serological tests often reveal low complement levels and the presence of a range of autoantibodies, including antinuclear antibody (ANA), anti-double-stranded DNA antibody (anti-dsDNA), and anti-Smith antibody (anti-Sm) positivity. SLE incidence is heavily skewed by gender, most often affecting women of childbearing age, with a female-to-male ratio as high as 9:1 [3,4].

The pathogenesis of SLE remains incompletely understood. The onset of SLE is generally believed to result from a disruption of immune homeostasis in genetically susceptible individuals triggered by specific environmental exposures, such as ultraviolet-B radiation, infections, and toxins. These environmental factors act as the initial trigger for abnormal autoimmunity by disrupting immune tolerance [5]. Due to immunological dysregulation and the effects of immunosuppressive therapy, SLE patients are susceptible to infections, which represent one of the leading causes of mortality in this patient population [6]. Nearly 37% of SLE patients experience at least one severe infection during the course of their disease [7], with respiratory infections being the most common [8]. Bloodstream infection (BSI) incidence is particularly detrimental, as such infections can trigger disease flares, prolong hospitalization, and increase medical costs, while also potentially leading to severe lupus flares, significantly elevating mortality risk. This risk is particularly prominent in the early stages of the disease [9,10]. Therefore, BSI incidence poses a serious threat to SLE patients, with implications for mortality risk, disease control, medical burden, and long-term prognosis, necessitating heightened attention in clinical prevention and management.

This study sought to analyze the distribution of pathogenic bacteria and clinical predictors for SLE-BSI systematically with the goal of providing a basis for early identification of high-risk patients.

## Materials and methods

### Study design and patient selection

This study retrospectively enrolled 215 SLE patients who fulfilled either the 2019 American College of Rheumatology (ACR) classification criteria for SLE [11] or the 2012 Systemic Lupus International Collaborating Clinics (SLICC) classification criteria [12] at the First Affiliated Hospital of Sun Yat-sen University between October 2013 and June 2023. The study included 43 SLE-BSI patients, with 172 SLE-non-BSI patients matched at a 1:4 ratio by age and sex, randomly selected from concurrent SLE inpatients in the same ward. The diagnosis of BSI required meeting both of the following criteria: (1) positive blood culture; and (2) the presence of at least one clinical manifestation suggestive of systemic infection, including body temperature <36 °C or ≥38 °C, chills, hypotension, heart rate >90 bpm, respiratory rate >20 breaths/min, or altered mental status [13]. Exclusion criteria included comorbid autoimmune diseases, malignancy, pregnancy or lactation, drug-induced lupus, or other active infections. The study protocol was approved by the Institutional Ethics Committee of The First Affiliated Hospital of Sun Yat-sen University ([2025] 146) and was conducted in accordance with the Declaration of Helsinki. The requirement for informed consent was waived due to the retrospective nature of the study.

### Data collection

Demographic data and clinical characteristics of the patients were collected, including age at sample collection, sex, underlying diseases, clinical symptoms, and clinical medication. To ensure clinical relevance, all data were retrospectively collected from medical records obtained within 24 h of admission, prior to blood culture collection. The following laboratory parameters were gathered: pathogenic species identified in blood culture, urinalysis findings including red and white blood cell counts, and the presence of autoantibodies, including ANA, anti-dsDNA, anti-Sm, anti-complement 1q (C1q) antibody, antiphospholipid (aPL) antibodies, perinuclear anti-neutrophil cytoplasmic antibody (P-ANCA), myeloperoxidase anti-neutrophil cytoplasmic antibody (MPO-ANCA), and proteinase 3 anti-neutrophil cytoplasmic antibody (PR3-ANCA). Levels of immunoglobulin A (IgA), immunoglobulin M (IgM), and immunoglobulin G (IgG), complement 3 and 4 (C3 and C4) levels, levels of inflammatory markers such as C-reactive protein (CRP), erythrocyte sedimentation rate (ESR), white blood cell (WBC) count, lymphocyte count, procalcitonin (PCT) levels, serum amyloid A (SAA) levels, and hematological parameters such as hemoglobin (HGB) and platelet (PLT) count were collected. Additionally, albumin (ALB) levels, 24-hour urine protein (24h urine protein) results, creatinine (CREA) levels, and Coombs test results were evaluated.

Pathogen identification in blood cultures was achieved using the BacT/ALERT VIRTUO automated blood culture system (bioMérieux, Shanghai, China). Urinalysis was performed using the LabUMat2 and iRised urine analysis system (Langmai, Guangzhou, China). ALB, CREA, and 24h urine protein were measured using the Beckman Coulter AU5800 series fully automated biochemical analyzer (Beckman Coulter, Suzhou, China). CRP and SAA were detected using the Mindray CRP-M100 (Mindray, Shenzhen, China), while complete blood count was analyzed using the Mindray BC-6800 plus analyzer (Mindray, Shenzhen, China). ESR was measured using the TEST1/THL instrument (Alifax, Shanghai, China). PCT was detected using the Roche cobas 8000 e801 analyzer (Roche, Shanghai, China). IgG, IgM, IgA, C3, and C4 levels were measured using a Siemens BNII system (Siemens, Shanghai, China). ANA and anti-C1q levels were determined using the Kaeser 6600 chemiluminescence immunoassay analyzer (Kangrun Biotech, Guangzhou, China). Anti-dsDNA was assayed using the iFlash 3000 chemiluminescence immunoassay analyzer (YHLO, Shenzhen, China). Anti-Sm was assayed using the MLIA-800 PLUS fully automated immunoassay analyzer (Lizhu Group, Zhuhai, China). MPO-ANCA, PR3-ANCA, aCL-IgM, and aCL-IgG were analyzed using the BIO-FLASH automated chemiluminescence immunoassay analyzer (Werfen, Beijing, China). P-ANCA was detected using the EURO Pattern automated immunofluorescence nuclear pattern and titer interpretation system (Euroimmun, Germany).

### Statistical analysis

Statistical analysis was performed using SPSS 27.0. Categorical variables were compared using the Chi-square test or Fisher’s exact test. The normality of continuous variables was assessed using the Shapiro-Wilk test. Comparisons of normally distributed continuous variables were conducted using Student’s t-test, while the Mann-Whitney U test was used for comparisons of non-normally distributed continuous variables. A *p* value of less than 0.05 was considered statistically significant. Variables showing a significant potential association with SLE-BSI (*p* < 0.05) in the univariate logistic regression analysis were included in the multivariate logistic regression model to identify independent risk factors for SLE-BSI.

## Results

### Pathogen distribution in the SLE-BSI group

Among the 43 SLE-BSI patients included in this study, 17 distinct pathogenic microorganisms were isolated and identified. Bacterial infections predominated (97.7%), with only one case of fungal infection (2.3%) identified as *Candida albicans*. Gram-positive bacteria accounted for the highest proportion (55.8%), with *Staphylococcus aureus* (*S. aureus*) being the most common species (18.6%). Gram-negative bacteria constituted the second largest group (41.9%), primarily *Salmonella spp.* (16.3%) and *Escherichia coli* (*E. coli*) (11.6%) (Table 1).

**Table 1.** Pathogen distribution in the SLE-BSI group.

Category of Pathogen	Major Pathogens	% (n/N)
Gram-positive bacterium	*Staphylococcus aureus*	18.6 (8/43)
	*Streptococcus agalactiae*	7.0 (3/43)
	*Staphylococcus epidermidis*	7.0 (3/43)
	*Listeria*	4.7 (2/43)
	*Gram-positive bacilli*	4.7 (2/43)
	*Streptococcus pneumoniae*	2.3 (1/43)
	*Viridans streptococci*	2.3 (1/43)
	*Staphylococcus cohnii*	2.3 (1/43)
	*Streptococcus oralis*	2.3 (1/43)
	*Staphylococcus lugdunensis*	2.3 (1/43)
	*Staphylococcus hominis subsp. Hominis*	2.3 (1/43)
Gram-negative bacterium	*Salmonella spp.*	16.3 (7/43)
	*Escherichia coli*	11.6 (5/43)
	*Pseudomonas aeruginosa*	9.3 (4/43)
	*Aggregatibacter actinomycetemcomitans*	2.3 (1/43)
	*Acinetobacter baumannii*	2.3 (1/43)
Fungus	*Candida albicans*	2.3 (1/43)

### Analysis of clinical data in SLE patients with and without BSI

The incidence of fever (86.0% vs. 60.5%, *p* = 0.002), fatigue (30.2% vs. 16.9%, *p* = 0.048), myalgia (14.0% vs. 2.9%, *p* = 0.010), headache (16.3% vs. 5.2%, *p* = 0.022), and seizures (7.0% vs. 0%, *p* = 0.008) was significantly higher in the SLE-BSI group compared to the SLE-non-BSI group (Table 2).

**Table 2.** Clinical and demographic data in the SLE-BSI and SLE-non-BSI groups.

Variables	SLE-BSI (*N* = 43) % (n/N) or median (IQR)	SLE-non-BSI (*N* = 172) % (n/N) or median (IQR)	*p*
Age (years)	28 (22–42)	27 (21–42.75)	0.790
Gender, female	79.1 (34/43)	81.4 (140/172)	0.728
Never smoked	97.7 (42/43)	98.8 (170/172)	0.490
Never drank alcohol	97.7 (42/43)	99.4 (171/172)	0.361
Hypertension	39.5 (17/43)	34.9 (60/172)	0.569
Diabetes mellitus	4.7 (2/43)	4.7 (8/172)	1.000
Coronary heart disease	0.0 (0/43)	1.2 (2/172)	1.000
Clinical manifestation			
Fever	86.0 (37/43)	60.5 (104/172)	0.002*
Fatigue	30.2 (13/43)	16.9 (29/172)	0.048*
Weight loss	14.0 (6/43)	9.9 (17/172)	0.418
Rash	16.3 (7/43)	15.1 (26/172)	0.850
Photosensitivity	2.3 (1/43)	0.6 (1/172)	0.361
Alopecia	9.3 (4/43)	5.2 (9/172)	0.298
Oral/nasal ulcers	9.3 (4/43)	4.7 (8/172)	0.264
Morning stiffness	2.3 (1/43)	1.7 (3/172)	1.000
Arthralgia	14.0 (6/43)	16.3 (28/172)	0.709
Arthritis	0.0 (0/43)	0.6 (1/172)	1.000
Muscle weakness	0.0 (0/43)	0.6 (1/172)	1.000
Myalgia	14.0 (6/43)	2.9 (5/172)	0.010*
Headache	16.3 (7/43)	5.2 (9/172)	0.022*
Seizures	7.0 (3/43)	0.0 (0/172)	0.008*
Chest pain	2.3 (1/43)	2.9 (5/172)	1.000
Pleural effusion	18.6 (8/43)	30.8 (53/172)	0.112
Pericarditis	0.0 (0/43)	1.2 (2/172)	1.000
Lupus nephritis	72.1 (31/43)	69.2 (119/172)	0.710
Clinical Medication			
Glucocorticoids	86.0 (37/43)	69.8 (120/172)	0.031*
Immunosuppressants	51.2 (22/43)	42.4 (73/172)	0.303
Intravenous immunoglobulin	51.2 (22/43)	27.3 (47/172)	0.003*
Antimalarials	60.5(26/43)	35.5 (61/172)	0.003*
Carbapenems	25.6(11/43)	15.1 (26/172)	0.104
Cephalosporins	0.0(0/43)	1.7 (3/172)	1.000
β-lactamase inhibitor combinations	18.6 (8/43)	27.3 (47/172)	0.241
Quinolones	9.3(4/43)	13.4 (23/172)	0.471
Glycopeptides	7.0(3/43)	2.3 (4/172)	0.145
Antifungal agents	2.3 (1/43)	5.2 (9/172)	0.691
Others	11.6 (5/43)	8.1 (14/172)	0.547

* *p* < 0.05. SLE, systemic lupus erythematosus; BSI, bloodstream infection.

The utilization rates of glucocorticoids (86.0% vs. 69.8%, *p* = 0.031), intravenous immunoglobulin (51.2% vs. 27.3%, *p* = 0.003), and antimalarials (60.5% vs. 35.5%, *p* = 0.003) were significantly higher in the SLE-BSI group than in the SLE-non-BSI group. There was no statistically significant difference in the use of antimicrobial agents between patients in the SLE-BSI group and those in the SLE-non-BSI group.

### Analysis of laboratory indices in SLE patients with and without BSI

Compared with the SLE-non-BSI group, the SLE-BSI group exhibited significantly higher average urinalysis white blood cell counts (median 42.20 cells/μL vs. 19.00 cells/μL, *p* = 0.001) and procalcitonin levels (median 0.33 ng/mL vs. 0.25 ng/mL, *p* = 0.006). The SLE-BSI and SLE-non-BSI groups did not differ significantly in terms of the autoantibody profile (including ANA, anti-dsDNA, anti-Sm, aCL-IgG, and aCL-IgM) or the levels of complement components C3 and C4 (all *p* > 0.05) (Table 3).

**Table 3.** Laboratory indices in the SLE-BSI and SLE-non-BSI groups.

Variables	SLE-BSI (*N* = 43) % (n/N) or median (IQR)	SLE-non-BSI (*N* = 172) % (n/N) or median (IQR)	*p*
WBC (×10^9^/L)	7.88 (4.45–13.71)	6.44 (4.09–9.75)	0.100
Lym(×10^9^/L)	0.46(0.29–1.14)	0.67 (0.42,1.10)	0.090
HGB (g/L)	92.00 (68.00–105.00)	94.00 (76.00–107.75)	0.090
PLT (×10^9^/L)	138.00 (81.00–223.00)	169.00 (109.25–230.50)	0.113
C3 (g/L)	0.50 (0.32–0.69)	0.53 (0.38–0.73)	0.254
C4 (g/L)	0.13 (0.07–0.20)	0.14 (0.08–0.21)	0.558
24h urine protein (g/24h)	1.88 (1.24–3.25)	1.88 (0.90–3.65)	0.143
URBC (cells/μL)	51.30(5.00–283.50)	22.20 (5.00–95.00)	0.086
UWBC (cells/μL)	42.20(11.10–150.50)	19.00 (6.33–39.25)	0.001*
CRP (mg/L)	8.59 (5.45–62.30)	8.59 (3.50–18.05)	0.312
ESR (mm/h)	32.00 (23.00–48.00)	32.00 (16.00–46.00)	0.561
ALB (g/L)	27.36 (20.60–30.10)	27.36 (23.13–32.08)	0.236
CREA (μmol/L)	92.00 (72.00–255.00)	83.50 (56.25–159.75)	0.106
IgA (g/L)	1.88 (1.08–2.26)	1.88 (1.36–2.65)	0.401
IgM (g/L)	0.60 (0.38–0.79)	0.61 (0.43–0.96)	0.154
IgG (g/L)	11.80 (7.15–16.50)	11.80 (8.12–15.65)	0.683
PCT (ng/mL)	0.33(0.23–0.77)	0.25 (0.11–0.49)	0.006*
SAA (mg/L)	51.50(32.70–283.00)	51.50 (23.25–137.00)	0.136
ANA	84.2 (32/38)	92.8 (141/152)	0.114
Anti-dsDNA	50.0 (19/38)	66.0 (101/153)	0.068
Anti-Sm	29.4 (10/34)	26.0 (34/131)	0.685
P-ANCA	14.3(4/28)	15.5 (18/116)	1.000
MPO-ANCA	3.6 (1/28)	4.3 (5/116)	1.000
PR3-ANCA	0.0 (0/28)	0.9 (1/116)	1.000
aCL-IgM	2.9(1/35)	8.7 (11/127)	0.465
aCL-IgG	11.4(4/35)	6.3 (8/127)	0.292
Coombs test	25.8 (8/31)	28.7 (31/108)	0.752

* *p* < 0.05. SLE: systemic lupus erythematosus; BSI: bloodstream infection; WBC: white blood cell; Lym: lymphocyte; HGB: hemoglobin; PLT: platelet; C3: complement 3; C4: complement 4; 24h Urine Protein: 24-hour Urine Protein; CRP: C-reactive protein; ESR: erythrocyte sedimentation rate; ALB: albumin; CREA: creatinine; IgA: immunoglobulin A; IgM: immunoglobulin M; IgG: immunoglobulin G; PCT: procalcitonin; SAA: serum amyloid A; ANA: antinuclear antibody; anti-dsDNA: anti-double-stranded DNA antibody; Anti-Sm: anti-Smith antibody; P-ANCA: perinuclear anti-neutrophil cytoplasmic antibody; MPO-ANCA: myeloperoxidase anti-neutrophil cytoplasmic antibody; PR3-ANCA: proteinase 3 anti-neutrophil cytoplasmic antibody; aCL-IgM: anti-cardiolipin IgM antibody; aCL-IgG: anti-cardiolipin IgG antibody.

### Independent clinical predictors associated with SLE-BSI

Univariate logistic regression analysis revealed that fever, myalgia, headache, and glucocorticoid use may offer value as predictors of SLE-BSI. Multivariate logistic regression analysis confirmed that fever (OR = 4.079, 95% CI: 1.582–10.517, *p* = 0.004), myalgia (OR = 5.637, 95% CI: 1.363–23.318, *p* = 0.017), and glucocorticoid use (OR = 2.690, 95% CI: 1.041–6.948, *p* = 0.041) were independent clinical predictors associated with the occurrence of BSI in SLE patients (Table 4).

**Table 4.** Independent clinical predictors associated with SLE-BSI.

Variables	Univariate Logistic Regression			Multivariate Logistic Regression		
	β	*p*	OR (95%CI)	β	*p*	OR (95%CI)
Fever	1.394	0.003*	4.032 (1.615–10.069)	1.406	0.004*	4.079 (1.582–10.517)
Myalgia	1.689	0.008*	5.416 (1.569–18.699)	1.729	0.017*	5.637 (1.363–23.318)
Headache	1.259	0.019*	3.522 (1.230–10.080)	0.975	0.095	2.651 (0.845-8.316)
Glucocorticoid use	0.983	0.037*	2.672 (1.063–6.718)	0.989	0.041*	2.690 (1.041–6.948)

* *p* < 0.05. SLE: systemic lupus erythematosus; BSI: bloodstream infection.

## Discussion

In this study, we characterized pathogen distribution and clinical predictors among patients with SLE-BSI. Stratification by Gram stain revealed that Gram-positive bacteria accounted for the majority of isolates, followed by Gram-negative organisms, a finding that contrasts with the report by Marcos et al. in which Gram-negative pathogens were more prevalent at 50.9% of isolates as compared with 43% for Gram-positive organisms [14]. At the species level, *S. aureus* emerged as the predominant Gram-positive pathogen, consistent with the previous study on SLE-BSI [14]. Among Gram-negative isolates, while most studies report *E. coli* as the most common species, followed by *Salmonella spp* [10,14–16], we observed no significant difference in detection rates between these two pathogens. These discrepancies may suggest regional variations in the pathogen distribution of SLE-BSI. The relatively high prevalence of *Salmonella spp.* aligns with the observation that SLE is an independent risk factor for *Salmonella* BSI, likely due to complement deficiency, impaired phagocytosis, and chronic immunosuppression [17,18].

SLE-BSI patients exhibited significantly higher urine white blood cell counts compared to SLE-non-BSI controls. Given that *E. coli* is the dominant uropathogen, this suggests that the urinary tract may be a potential source of bloodstream invasion in SLE patients. Therefore, in clinical practice, heightened vigilance for BSI development is warranted in SLE patients who develop urinary tract infections to facilitate early intervention. Beyond source identification, the interplay between bacterial infection and SLE pathogenesis involves shared immunological pathways. Bacterial components (e.g. LPS, CpG DNA) can activate plasmacytoid dendritic cells *via* Toll-like receptors, driving excessive type I interferon production, a central feature of SLE immunopathology [19]. Additionally, molecular mimicry or superantigen-mediated activation of autoreactive T cells may further breach immune tolerance [20,21]. Thus, BSI in SLE may not merely be a complication of immunosuppression but also a potential trigger for disease flares, underscoring the bidirectional relationship between infection and autoimmunity.

We observed no significant differences between SLE-BSI patients and SLE-non-BSI controls in terms of SLE-associated autoantibody profiles. This finding suggests the development of BSI may not be primarily driven by heightened lupus-specific humoral autoimmunity or conventional serological markers of disease activity. Rather, this may suggest that factors external to the core autoimmune process, such as immunosuppressive therapy, disruption of mucosal or skin barriers, and increased exposure to pathogens, could play a more prominent role in influencing susceptibility to BSI among patients with SLE.

Although fever and myalgia are common manifestations of BSI, myalgia is also a common feature during SLE flares [7]. In this retrospective cohort, we found that SLE-BSI patients were more likely to present with fever and myalgia, which served as independent clinical predictors. As manifestations of the systemic inflammatory response, the combination of fever and myalgia offers a valuable indicator for identifying potential BSI in SLE patients. Given that SLE disease activity can often mimic infectious symptoms, new-onset or worsening myalgia, especially when associated with fever, should prompt suspicion of BSI. Therefore, clinicians could utilize these combined indicators to guide timely blood culture testing and empirical treatment.

Moreover, glucocorticoid use was identified as another significant independent clinical predictor associated with the occurrence of BSI in this study. The underlying mechanism may stem from the potent anti-inflammatory and multifaceted immunosuppressive effects of glucocorticoids, including reduced expression of cytokines and adhesion molecules, the inhibition of leukocyte migration and their ability to migrate to sites of inflammation, and interference with the functions of leukocytes, fibroblasts, and endothelial cells [22]. High-dose glucocorticoids (e.g. prednisolone ≥60 mg/day) can induce a more profound state of immunosuppression, thereby increasing susceptibility to pathogen exposure and elevating the risk of BSI [23]. Simultaneously, long-term glucocorticoid use further elevates the risk of BSI [24,25], suggesting the need for clinical vigilance when monitoring SLE patients receiving glucocorticoid therapy.

This study has several limitations. First, its retrospective, single-center design resulted in findings that may be influenced by center-specific factors, including regional demographics, SLE phenotypes, treatment protocols, and microbial epidemiology. Second, the potential for residual confounding is inherent in this retrospective study. The 10-year study period introduces potential bias from evolving treatment landscapes, infection control practices, and diagnostic criteria. To minimize the impact if these factors, we employed strict selection criteria, 1:4 matching, and multivariable regression. In addition, granular data on glucocorticoid exposure, such as peak doses and cumulative intake, were incomplete for some patients due to cross-institutional care. Although we employed a simplified classification to maintain statistical power, this limited a detailed analysis of dose-response relationships. Future large-scale, multicenter prospective cohorts are needed to validate the clinical predictors for SLE-BSI and elucidate the dynamic association between glucocorticoid patterns and BSI risk, thereby guiding precision prevention in SLE.

## Conclusion

The results of this study highlight *Staphylococcus aureus*, *Escherichia coli*, and *Salmonella spp.* as the primary pathogens associated with BSI incidence in SLE patients. In addition, fever, myalgia, and glucocorticoid use were identified as independent clinical predictors for BSI. Thus, these factors should be assessed systematically in the comprehensive clinical management of SLE patients with BSI. For SLE patients on glucocorticoid therapy, especially when myalgia and fever are present, the utility of blood culture testing may be considered, thereby facilitating the early identification of BSI.

## Data Availability

Data will be available upon reasonable request from the corresponding author (Liubing Li, Email: lilb8@mail.sysu.edu.cn).
